# Outcome of Laparoscopic Repair for Perforated Peptic Ulcers in a Resource-Limited Setting

**DOI:** 10.7759/cureus.24159

**Published:** 2022-04-15

**Authors:** Mahmood Ayyaz, Ahsan Shafiq, Usman Ismat Butt, Wasim H Khan, Muhammad Umar, Ali Abaid

**Affiliations:** 1 Surgery, National Hospital and Medical Centre, Lahore, PAK; 2 Surgical Oncology, Shaukat Khanum Memorial Cancer Hospital and Research Centre, Lahore, PAK; 3 Surgery, Services Hospital, Lahore, PAK; 4 Surgery, Hayat Memorial Teaching Hospital, Lahore, PAK

**Keywords:** resource limited, outcome, emergency, perforated peptic ulcers, laparoscopic repair

## Abstract

Background

Perforation of peptic ulcers is a common cause of emergency surgery and has significant morbidity and mortality. The use and range of laparoscopic surgery have greatly increased over the past three decades. Laparoscopic approach is an option for perforated peptic ulcers because of the simple nature of the intervention. The aim of this study was to evaluate the outcome of laparoscopic approach for peptic ulcer repair in emergency setting by means of operative time, post-operative pain, mean hospital stay, and post-operative complications.

Methods

In this study, we enrolled patients presenting with perforated peptic ulcers in the emergency department of a tertiary care hospital in Lahore, Pakistan. Approval from the hospital ethical committee and informed consent were taken from all patients. After resuscitation, the patient underwent laparoscopic repair of perforation. Post-operative course of patients was monitored. Duration of surgery, post-operative pain, length of hospital stay, and post-operative complications were noted for all patients.

Results

Between December 2018 and December 2021, 31 patients with perforated peptic ulcers underwent laparoscopic repair at our hospital. Mean age of patients was 37.25 ± 7.80 years. Most of the patients were male (70.76%). The mean operation time was 109.35 ± 17.02 minutes for laparoscopic repair. Mean duration of hospital stay was 5.10 ± 0.87 days. Mean post-operative pain was 3.55 ± 0.85 assessed using the Visual Analogue Scale. There were no mortalities during the 30-day post-operative window.

Conclusion

With proper patient selection, laparoscopic surgery offers better results as compared to open surgery in patients undergoing emergency surgery for perforated peptic ulcers.

## Introduction

An ulcer is a break in the epithelial lining, while a peptic ulcer constitutes a breach in the mucosa of the stomach or duodenum. It occurs as a result of the mismatch between the protective mechanisms and acid secretion [1]. It is usually extremely painful. One of the most common causative agents of it is *Helicobacter pylori*. Other causative or aggravating factors include alcohol, smoking, and drugs such as non-steroidal anti-inflammatory drugs (NSAIDs) [2]. The lifetime prevalence of peptic ulcer disease in the general population is 5-10% [3]. It is a commonly encountered problem in third-world countries like Pakistan [4]. Although there has been a reduction in its incidence in the last three decades due to extensive usage of proton pump inhibitors but complications are still seen in 10-20% of cases [5].

The lifetime prevalence of perforation in patients with peptic ulcer disease (PUD) is about 5%. Despite advances in management, the incidence of peptic ulcer perforation has not decreased and emergency surgery still has to be performed to deal with the complications of peptic ulcer disease [6]. Perforation is the most common cause of emergency operation and a mortality of 30% has been reported in such cases [7]. Depending on hemodynamic instability, co-morbid conditions, duration of symptoms, and history of chronic ulcer disease different surgical approaches are used. The most common of them is Graham patch repair which can either be done using a laparoscope or by doing a laparotomy [8].

The use and range of laparoscopic surgery have greatly increased over the past three decades. Laparoscopic approach is an option for perforated peptic ulcers because of the simple nature of the intervention. Identification of the perforation, peritoneal lavage, and perforation closure all can be done by laparoscopic approach while avoiding the large incision and associated complications of the open approach. Mouret is credited with the first laparoscopic intervention for perforated duodenal ulcers in 1990 [9]. Laparoscopic surgery due to its many advantages is fast becoming the preferred approach in most situations. However, studies comparing open with laparoscopic management of peptic ulcer perforation have produced mixed results [10]. Laparoscopic surgery has been in practice in our country for almost three decades but the availability of laparoscopy in emergency setting is still limited. We chose to evaluate the outcomes of laparoscopic approach for peptic ulcer repair in the emergency department in a resource-limited setting. The objective of the study was to evaluate the outcomes of the laparoscopic repair of peptic ulcer perforation in terms of mean operative time, mean hospital stay, mean postoperative pain, and complications.

## Materials and methods

Between December 2018 and December 2021, patients who presented with peptic ulcer perforation in the emergency department were enrolled in the study. A single-center prospective observational study was conducted at our hospital. Any patients younger than 20 years or older than 70 years, duration of symptoms more than 48 hours, having unstable vitals after resuscitation, having a history of previous abdominal surgery or peritoneal dialysis, or refusing to participate in the study were excluded. Procedure was explained in detail to the family and patient. Benefits and possible complications were discussed. Informed consent was obtained from all patients before surgery. Approval from the Institutional Review Board Services Institute of Medical Sciences (ref. no.: IRB/2018/443/SIMS) was taken prior to the start of the study.

Patients who had presented with suspected peptic ulcer perforation were included in the study. Patient demographic information including age, gender, and BMI was noted. Diagnosis of peptic ulcer was suspected on the basis of clinical history and examination. History suggestive for peptic ulcer perforation included epigastric pain, intake of NSAIDs, previous diagnosis of acid peptic disease, or intake of proton pump inhibitors. Clinical examination suggestive of perforated peptic ulcer disease included tachycardia, fever, and tenderness in the epigastrium or abdomen. Abdominal and chest x-ray was used in all cases to confirm the presence of free air under the diaphragm. All the patients were resuscitated with intravenous fluids and antibiotics during the preoperative period. Nasogastric aspiration for stomach decompression and urethral catheterization for monitoring output was done in every patient. Such cases after informed consent and stabilization underwent diagnostic laparoscopy under general anesthesia in reverse Trendelenburg position. Any patients found to have etiology other than peptic ulcer perforation on diagnostic laparoscopy were excluded from the study. After thorough lavage to clear intra-peritoneal contents and spillage, laparoscopic Graham patch repair was done. We made use of omentum to plug the defect. Suture was placed on either of the perforations to hold the omentum in place. A drain was placed in the right subhepatic space next to the repair in all cases, which was removed after 48 hours. All patients were shifted toward post-operative care till discharge. Same post-operative management was done in all patients. Patients were mobilized on the second post-operative day and Foley’s catheter was removed. All patients were kept nil per oral for four days during which partial parenteral nutrition was provided. Patients were allowed orally on the fourth post-operative day upon which the nasogastric tube was also removed.

Duration of the procedure was noted which was considered to be from the induction of anesthesia to the extubation of patients. Post-operatively, pain was assessed at 6, 12, and 24 hours using the Visual Analogue Scale. Length of hospital stay was noted for all patients. Post-operative complications and 30-day mortality were also noted. Comparison with previously documented incidence was also done.

## Results

This study was carried out between December 2018 and December 2021. Thirty-one consenting patients fulfilling the inclusion criteria were included in the study. The mean age of the patients was 37.25 ± 7.80 years. Twenty-two patients were male (70.96%) and nine patients (29.03%) were female. The mean operative time was 109.35 ± 17.02 minutes for laparoscopic repair. The mean duration of hospital stay was 5.10 ± 0.87 days. The mean post-operative pain score was 3.55 ± 0.85, which was assessed using the Visual Analogue Scale (Table 1).

**Table 1:** Summary of results of the patients who fulfilled the inclusion criteria

Variable	Mean± SD
Age (in years)	37.25 ± 7.80
BMI (kg/m^2^)	28.87 ± 2.66
Duration of surgery (min)	109.35 ± 17.02
Duration of hospital stay (days)	5.10 ± 0.87
Mean post-operative pain	3.55 ± 0.85

None of our patients had to be converted to open during this study. Four patients had surgical site infection of which three were superficial and settled with conservative management. One patient had a deep surgical site infection. He developed an intra-abdominal abscess in the right subhepatic space which required ICU admission as well as a re-look surgery. There were no mortalities during the 30-day post-operative window (Table 2). 

**Table 2:** Break-down of complications SSI: surgical site infection

Variable	Number (%)
Conversion	0
SSI	4
Superficial SSI	3 (9.67%)
Deep SSI	1 (3.22%)
ICU admission	1 (3.22%)
Deep venous thrombosis/pulmonary embolism	0
Acute kidney injury	1 (3.22%)
Post-operative leak (clinical assessment)	0
30-day mortality	0

## Discussion

In our study, we found that the laparoscopic approach in selected patients with peptic ulcer perforation provides results comparable to those reported for open surgery in emergency setting. The mean duration of surgery in our study was similar to the ones reported by others. As has been concluded by various studies laparoscopic approach results in small incisions which have an effect of decreased post-operative pain. The mean pain score in our study as evaluated by the Visual Analogue Scale was 3.55 ± 0.85. Although the duration of hospital stay in our cases was almost five days, this is partially due to the fact that we kept our patients nil per oral (NPO) for 96 hours as per convention. Most of the patients were mobile and out of bed on the second post-operative day with removal of drain and nasogastric tube. Partial parenteral support was continued for four days at which patients were allowed orally and discharged after they tolerated oral intake. There was no mortality among our patients. There was no conversion in our study. Morbidity was seen in four cases.

In the study done in 2015 by Wong et al., operating time for laparoscopic repair was 145±19 minutes and for open repair, it was 110±13 minutes [11]. Mean post-operative pain (first 24 hours) was 4.4±0.8 vs 7.0±0.9, respectively. The mean hospital stay was 6.9±2.2 vs 8.9±3.3 days. The mean hospital stay and pain score in our study were 5.10 days and 3.55, respectively, which are comparable to the results obtained. In the review carried out by Lunevicius and Morkevicius, it was concluded that for low-risk patients, laparoscopic repair seemed a better option than open repair [12]. However, due to limited data on laparoscopic approach, open repair should be considered especially in high-risk patients. By selecting patients who had stable vitals after initial resuscitation our results were similarly favorable. In a study by Siow et al., 131 cases reported that laparoscopic repair resulted in reduced wound infection rates, shorter hospitalization, and reduced postoperative pain as compared to open repair [13]. Matsuda et al. showed that although a level of expertise in laparoscopic skills is needed, surgeons familiar with basic laparoscopic procedures such as cholecystectomy readily pick up the required skills and perform surgery after some practice [14]. A number of meta-analyses have been done on the subject. Initial meta-analysis done before 2010 usually didn’t find one technique superior to the other [15]. However, with the progression of time, a recently done meta-analysis has shown that the laparoscopic repair leads to decreased post-operative analgesic requirement, shorter hospital stay, a lower risk of wound infection, and a lower mortality rate as compared to the open approach but with similar morbidity, mortality, and reoperation rates [12,16,17].

It was demonstrated by Zhou et al. that omental patches offered no additional benefits which was also concluded by others [17]. There is some variability in surgical technique when performing repair of ulcer perforation. The simple closure requires less operative time as compared to omental patches or overlay [8]. It was further shown that the more experience gained by the surgeon the shorter the operative time became. Performing a thorough intra-abdominal lavage and intra-corporeal knotting might be time-consuming, especially for the novice laparoscopic surgeon. Operative time can vary depending upon the amount of fluid used for irrigation as well as on the suction device [18].

The main limitation of our study is the small sample size, selection bias, and no comparison to another open surgery group. Although it extended over three years, finding patients who fit the inclusion criteria was challenging. The strict inclusion criteria were however kept to ensure the safety of the patients. Furthermore, it was somewhat difficult to carry out laparoscopic surgeries in the emergency setting due to constraints of manpower and resources. Still, our study has shown that laparoscopic repair for selected cases of perforated peptic ulcers yields good results with no added morbidity. We believe it is one of the first studies to be carried out in this part of the world. The results of our study should encourage further usage of laparoscope in such cases.

## Conclusions

Peptic ulcer disease is a common pathology. Despite advances in the management of peptic ulcer disease, many patients still present with complications. Perforated peptic ulcer is a serious emergency that often requires surgical intervention and is associated with morbidity. Our study shows that, with proper patient selection, laparoscopic surgery offers results comparable to results reported in the literature regarding open surgery in patients undergoing emergency surgery for perforated peptic ulcers.
